# Rate and reasons for peritoneal dialysis dropout following haemodialysis to peritoneal dialysis switch: a systematic review and meta-analysis

**DOI:** 10.1186/s12882-024-03542-w

**Published:** 2024-03-16

**Authors:** Xingge Sun, Clare McKeaveney, Joanne Shields, Chi Peng Chan, Matthew Henderson, Fiona Fitzell, Helen Noble, Stephen O’Neill

**Affiliations:** 1 School of Nursing and Midwifery, Queen’s University Belfast, 97 Lisburn Rd, Belfast, BT9 7BL UK; 2 Regional Nephrology & Transplant Unit, Belfast City Hospital, 51 Lisburn Road, Belfast, BT9 7AB UK; 3 Centre for Medical Education, Queen’s University Belfast, Whitla Medical Building, Belfast, BT9 7BL UK

**Keywords:** Haemodialysis, Peritoneal dialysis, Dropout, Technique failure, Systematic review

## Abstract

**Background:**

Patient experiences and survival outcomes can be influenced by the circumstances related to dialysis initiation and subsequent modality choices. This systematic review and meta-analysis aimed to explore the rate and reasons for peritoneal dialysis (PD) dropout following haemodialysis (HD) to PD switch.

**Method:**

This systematic review conducted searches in four databases, including Medline, PubMed, Embase, and Cochrane. The protocol was registered on PROSPERO (study ID: CRD42023405718). Outcomes included factors leading to the switch from HD to PD, the rate and reasons for PD dropout and mortality difference in two groups (PD first group versus HD to PD group). The Critical Appraisal Skills Programme (CASP) checklist and the GRADE tool were used to assess quality.

**Results:**

4971 papers were detected, and 13 studies were included. On meta-analysis, there was no statistically significant difference in PD dropout in the PD first group (OR: 0.81; 95%CI: 0.61, 1.09; I^2^ = 83%; *P* = 0.16), however, there was a statistically significant reduction in the rate of mortality (OR: 0.48; 95%CI: 0.25, 0.92; I2 = 73%; *P* = 0.03) compared to the HD to PD group. The primary reasons for HD to PD switch, included vascular access failure, patient preference, social issues, and cardiovascular disease. Causes for PD dropout differed between the two groups, but inadequate dialysis and peritonitis were the main reasons for PD dropout in both groups.

**Conclusion:**

Compared to the PD first group, a previous HD history may not impact PD dropout rates for patients, but it could impact mortality in the HD to PD group. The reasons for PD dropout differed between the two groups, with no statistical differences. Psychosocial reasons for PD dropout are valuable to further research. Additionally, establishing a consensus on the definition of PD dropout is crucial for future studies.

**Supplementary Information:**

The online version contains supplementary material available at 10.1186/s12882-024-03542-w.

## Introduction

Patients with end-stage kidney disease (ESKD) require lifelong renal replacement therapy (RRT) via haemodialysis (HD), peritoneal dialysis (PD), or transplantation [1, 2]. Until 2021, 69,497 adult patients in the United Kingdom were undergoing RRT due to ESKD. Among them, 56% had undergone transplantation, 38% were receiving HD, and 6% had PD [3]. Notably, 49% of patients transfer to different dialysis modalities in the first year of dialysis due to treatment requirements, resources, and goals which may change over time [4–5]. Based on the Canadian Organ Replacement Register data, there were 53,493 patients started on RRT from 2001 to 2010. Among these, 3,757 patients (7%) who initially underwent HD switched to PD within the first year of HD [6].

Initiating dialysis is a traumatic experience for patients requiring psychological support [7, 8]. Additionally, the shift in dialysis modalities acts as an extra stressor for patients and negatively influences patients’ health-related quality of life as people must adapt to new circumstances [9, 10]. However, compared to HD, PD has more flexibility, less restrictions, lower risk of infection, better preservation of residual kidney function and is associated with lower healthcare costs than HD [11–13]. Despite this, more than 35% of patients drop out of PD and transfer to HD [14].

Qazi et al. [15] conducted a scoping review of various factors impacting dialysis withdrawal. They suggested that demographic factors, renal disease aetiology, health behaviour, comorbidities, dialysis indicators, and other individual factors will all impact dialysis withdrawal. Therefore, decision making around dialysis choices are complex and multi-factorial. Furthermore, a recent systematic review and meta-analysis compared the differences in outcomes for patients transferring from HD to PD and PD as initial therapy in eight published articles [16]. They suggested that HD to PD switch was associated with poorer outcomes, including inferior survival and technique outcomes, compared to patients who were PD first. However, this review only explored the reasons related to transferring from HD to PD. It is not clear why patients drop out of PD following a HD to PD switch. The previous review also excluded patients who had been on HD for < 90 days.

This study aims to assess the factors leading to the switch from HD to PD, rate and reasons for PD dropout and mortality difference following any time on HD prior to switching to PD. Identifying factors associated with PD dropout in HD to PD switch patients will inform clinical decision-making and establish support requirements for patients considered for HD to PD switch. In this study, PD dropout was defined as any PD patient who stopped PD for any reason other than death, kidney transplantation or a personal decision to stop all RRT (i.e., withdrawal from dialysis and transition to conservative or palliative care).

## Method

### Inclusion and exclusion criteria

The following inclusion criteria were applied to screening studies as a part of this systematic review: (1) Studies carried out on adults (age > 18) undergoing dialysis. (2) Studies comparing two groups including (i) patients on HD for any length of time who then switched to PD (HD to PD group) and (ii) patients started on PD as initial therapy (PD first). (3) Studies illustrating outcomes associated with the incidence or reasons for PD dropout and mortality. (4) Studies written in English. (5) Randomised controlled trials, non-randomised controlled trials, and observational studies (cohort and case-control studies). Exclusion criteria: (1) systematic reviews, posters, and conference abstracts. (2) Studies that did not report the outcomes associated with this systematic review. (3) Studies that failed to include initial data for the two groups or failed to divide the study sample into the appropriate groups. There were no restrictions on study settings, year of publication, or minimum follow-up period in this systematic review. The PRISMA (Preferred Reporting Items for Systematic Reviews and Meta-analyses) checklist was followed when reporting this review [17], and the research protocol was registered at PROSPERO (study ID: CRD42023405718).

### Search strategy

Four databases were used to detect potential articles, including Medline, PubMed, Embase, and Cochrane. The last search was run on March 8, 2023. An initial literature search using the following keywords in a varied order: “haemodialysis”, “peritoneal dialysis”, “PD”, “HD”, “transfer*”, “change”, “switch”, “shift”, “transition”, “dropout”, “drop-out”, “stop”, “withdraw” was performed. A librarian assisted with optimisation of the search. Associated references and sources were assessed after a review of reference lists and included as appropriate. Two reviewers (X.S. and C.M.) screened the titles and abstracts first. Full-text articles were screened based on the inclusion and exclusion criteria for further data extraction and quality assessment. Any disagreement was discussed and agreed upon with a third reviewer (S.O.).

### Data extraction

The tables of data extraction agreed upon by all reviewers was used to extract data, including: authors, publication year, study type, sample size, BMI, age, gender, cause of renal disease, time on HD, creatinine clearance, hemoglobin levels, albumin levels, urine output, follow-up period, the definition of technique failure, and study outcomes (the rate of PD drop out, reasons for PD drop out, mortality, and transplantation), and the reasons for HD switch to PD. The primary outcomes were dropout events, technique survival rates, and mortality rates in HD to PD groups compared to PD first groups. The secondary outcomes were the factors leading to the switch from HD to PD and reasons for PD dropout in HD to PD groups compared to PD first groups.

### Quality assessment

The Critical Appraisal Skills Programme (CASP) checklist for cohort studies was applied to assess the quality of the articles included. The CASP checklist includes three parts with 12 questions that are answered with “yes”, “can’t tell” and “no” [18]. The scores for these responses are 2 (yes), 1 (can’t tell), and 0 (no) [19]. To minimise bias two researchers (C.M., S.O.) scored a sample of the articles after they were initially scored by another researcher (X.S.). Additionally, GRADE profiler was used to rate the strength of overall evidence quality. In the GRADE system observational studies begin as low-quality evidence supporting estimates of intervention effects. This is followed by review of five factors (risk of bias, inconsistency, indirectness, imprecision, publication bias) to downgrade and three factors (large effect, dose-response, all plausible confounding) to upgrade the quality of evidence [20]. Ultimately, the quality level and evidence certainty were presented.

### Statistical analysis

Review Manager 5.4.1 was applied for meta-analysis. The total number of patients and events (PD dropout and mortality) in the PD first groups and HD to PD groups were extracted from the included studies. In a random-effects model, the Mantel-Haenszel odds ratio (OR) with 95% confidence intervals (CI) was used to summarise categorical data on PD dropout and mortality [16]. To calculate statistical heterogeneity, the I^2^ statistic was used. I^2^ levels of 25–50% were classified as low heterogeneity, values of 50–75% were classified as moderate heterogeneity, and values of more than 75% were classified as significant heterogeneity [16].

## Results

**Fig. 1:**
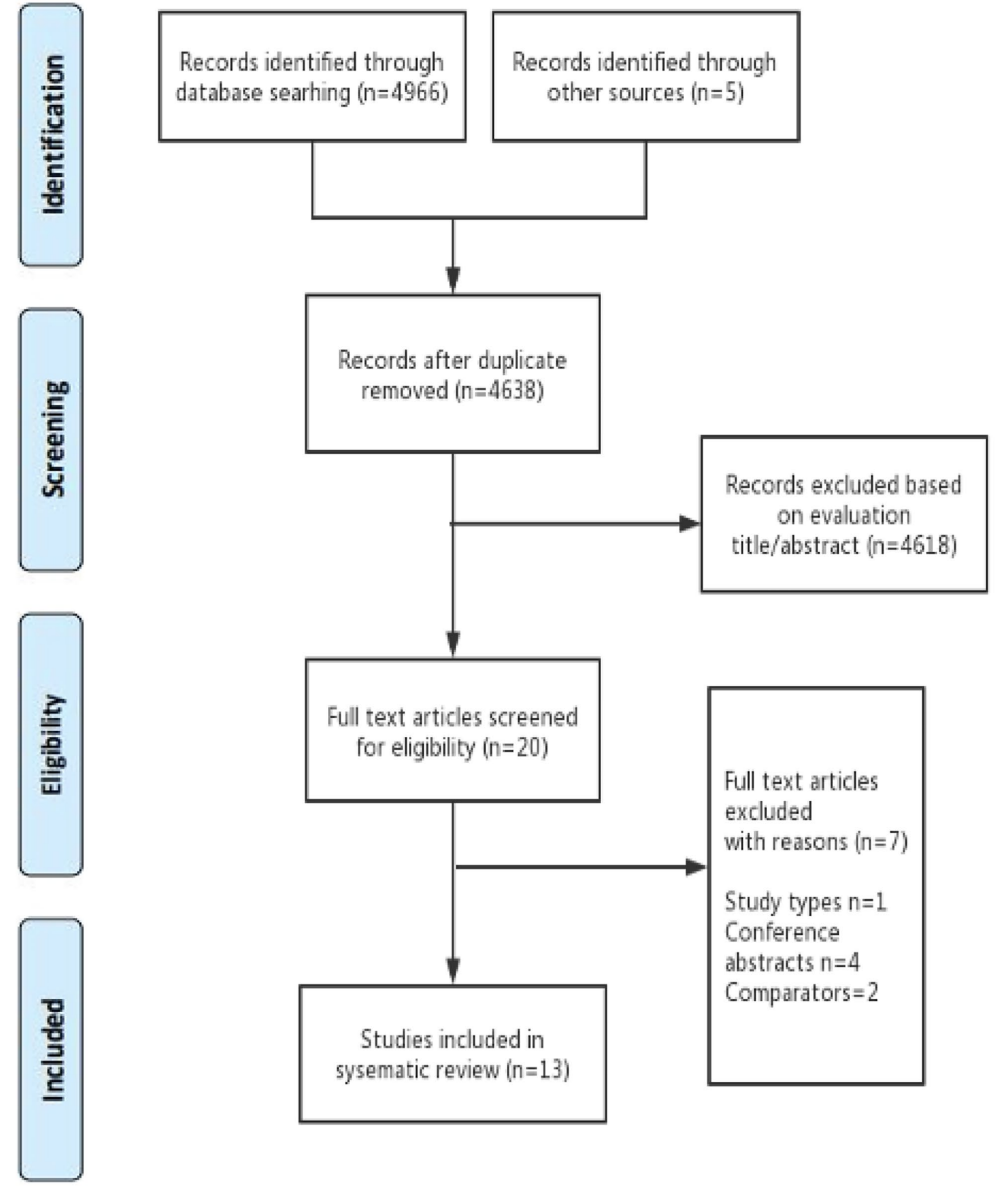
Study flow chart

4966 articles were identified from databases and five articles were screened from the reference lists. After removing duplicates and screening titles and abstracts, 20 studies were chosen for the full-text review. Ultimately, 13 articles were included in this systematic review [6, 21, 22–32]. Seven studies were excluded for the following reasons: one study was a systematic review [16], four studies were conference abstracts [33–36], in one study comparators were HD first and HD to PD group [37], and one study had a small sample size without comparators [38]. The detailed information relevant to the search results is presented in a study flow chart (**See** Fig. 1).

### Characteristics of patients in the included studies

The characteristics of patients including sample size, mean age, BMI, time on HD, diabetes as a cause of ESRD, creatinine clearance, urine output, haemoglobin, albumin, and the time of follow-up are shown in Table 1. Of the 13 included studies, 12 studies are cohort studies, and one study is a case-control study [31]. The sample sizes of the PD first group and HD to PD group range from 37 to 9404 and 28 to 3757 patients, respectively, and include patients from Argentina, Canada, China, Turkey, Poland, France, Iran, Australia, New Zealand, and America. Additionally, the duration of HD varied from less than a month to 286 months, and the follow-up time varied from one month to 132 months. Each group of studies had varying degrees of missing data that is summarised in Table 1.

**Table 1:** The characteristics of patients in included studies

Author Year	Groups	Sample size	Mean age (years)	BMI (Mean)	Male gender (%)	Median time on HD (months)	Diabetes as cause of ESRD (%)	Creatinine Clearance (L/wk/1.73m2) Mean	Urine output (ml/24 h) Mean	Hemoglobin (g/L) Mean	Albumin(g/L) Mean	Follow-up
Barone et al., 2014 [22]	PD first	37	52 ± 15	N	35	49 ± 45	N	72 ± 25	N	N	N	N
	HD-PD	73	52 ± 15	N	43		N	62 ± 19	N	N	N	N
Chidambaram et al., 2011 [23]	PD first	4206	62 ***	N	56**	≤ 3	N	N	N	N	N	2 ± 2 y (s)
	HD-PD	956		N			N	N	N	N	N	
Dong et al., 2022 [24]	PD first	854	N	N	N	≤ 3*	N	N	N	N	N	>1y
	HD-PD	1437	N	N	N		N	N	N	N	N	
Koc et al., 2012 [25]	PD first	251	49 ± 16*	N	46	33 ± 34 (3-144)	29	57 ± 18	389 ± 390*	105 ± 17*	36 ± 6	40 ± 26 m (s)
	HD-PD	48	44 ± 16	N	35		21	51 ± 13	244 ± 536	112 ± 22	37 ± 5	
Liberek et al., 2009 [21]	PD first	197	52 ± 15	24 ± 4	57*	18 (3-286)	28.4	N	1130 ± 768**	97 ± 12***	37 ± 7	20.5 m (s) (1-132)
	HD-PD	67	50 ± 15	23 ± 4	40			N	400 ± 410	106 ± 17	37 ± 5	
Lobbedez et al., 2012 [26]	PD first	7805	N	N	N	N	N	N	N	N	N	N
	HD-PD	1748	N	N	N	N	N	N	N	N	N	N
Lobbedez et al., 2013 [27]	PD first	7931	71	N	57	≤ 1	21	N	N	N	N	>1y
	HD-PD	568	69	N	57		23	N	N	N	N	
Najafi et al., 2012 [28]	PD first	1067	46 ± 21**	23 ± 5	42**	18 (3-284)	N	84 ± 33***	918 ± 693	N	36 ± 7	14 m (s) (3-119)
	HD-PD	245	48 ± 16	23 ± 4	36		N	64 ± 33	446 ± 473	N	36 ± 7	
Nessim et al., 2015 [6]	PD first	9404	N***	26(23–30)	58	83d (38–158)	39***	N	N	111(101–120)***	36 (32–40)***	>3 y (s)
	HD-PD	3757	N		60		36	N	N	97(86–110)	32 (27–37)	
Nguyen et al., 2019 [29]	PD first	1822	62***	N	57	6 (4–11)	36.2**	41 (34–49) ***	N	115 (105–126)	N	6 y (s)
	HD-PD	911	58	N	58		37.9	22 (5–53)	N	110 (98–112)	N	5 y (s)
Pulliam et al., 2014 [30]	PD first	1313	58 ± 15***	32 ± 11***	55	≤ 3	N	N	N	116 ± 15**	36 ± 5	>1 y
	HD-PD	364	55 ± 15	32 ± 12	59		N	N	N	113 ± 17	36 ± 5	
Zhang et al., 2013 [31]	PD first	66	64 (49–70)	23 ± 4	45	7 (3-187)	35	N	750(600–1250)**	92 ± 18	40 ± 5	18 m (s)
	HD-PD	33	65 (52–69)	20 ± 3	42		30	N	0 (0-775)	95 ± 19	37 ± 6	17 m (s)
Zhang et al., 2008 [32]	PD first	40	N	N	28	>3	N	60 ± 16	N	80 ± 14	35 ± 5*	>2 y (s)
	HD-PD	28	N	N	28		N	75 ± 24	N	86 ± 12	37 ± 4	

Abbreviations: HD, Hemodialysis; PD, Peritoneal dialysis; N, unknown Note: *=*p* < 0.05 **=*p* < 0.01 ***=*p* < 0.001

### The clinical outcomes of included articles

The summary of clinical outcomes (dropout events, median time on PD, technique survival rates, the reasons for PD dropout, mortality rates, transplantation rates, and technique failure (TF) definitions) are presented in Table 2. Reasons for HD switch to PD are reported in Table 3. Detailed explanations of the tables are as follows.

**Table 2:** The rate and reasons for patient’s dropout in PD

Author Year	Groups	The number of dropouts	Median time on PD	TS rates	Reasons for PD dropout	Mortality rates	Transplant- ation rates	The definition of TF
Nessim et al., 2015 [6]	PD first	3668	48 m(s)	HD-PD<PD *	Inadequate PD: 3% * Peritonitis: 3% * other causes: 12%	*n* = 3170 (24%) HD-PD > PD *	N	Transfer to HD for 90 days or more
	HD-PD	1597	36 m(s)		Inadequate PD: 4% Peritonitis: 4% other causes: 18%		N	
Liberek et al., 2009 [21]	PD first	N	N	HD-PD<PD *	Correlation between peritonitis and technique failure. Anuric patients: dialysis adequacy problems, especially related to volume status and overhydration	1year: 8% 2years: 24%	*n* = 100 (38%)	N
	HD-PD	N	N					N
Barone et al., 2014 [22]	PD first	N	57 ± 42 m(s)	1year:100% 3years:96% 5years:90%	N	1 year: 0% 3years: 10%	N	Transfer to HD or death
	HD-PD	N	51 ± 49 m(s)	1year:94% 3years:83% 5years:75%	N	1 year: 5% 3years:25%	N	
Chidambaram et al., 2011 [23]	PD first	*n* = 1323	N	1year:84% 2years:78% 5years:58%	N	*n* = 2798 (54%)	N	Transfer to HD for > 2 months.
	HD-PD		N		N		N	
Dong et al., 2022 [24]	PD first	80	N	1year: 93%	Mechanical failure and infection	4.7%	7.3%	Transfer to HD for > 30 days or death
	HD-PD	93	N					
Koc et al., 2012 [25]	PD first	80	N	N	Sepsis/peritonitis *n* = 50 (20%) Cardiovascular events *n* = 24 (10%) Malnutrition *n* = 3 (1%) Insufficiency *n* = 3 (1%)	*n* = 55 1year: 9% 2years:18%	*n* = 39 (16%)	N
	HD-PD	0**	N	N	N	*n* = 23 1year: 50% 2years: 59% **	*n* = 2 (42%)**	
Lobbedez et al., 2012 [26]	PD first	Total: *n* = 2464	17 m(s)	HD-PD<PD	Dialysis adequacy *n* = 612 (25%) Peritonitis *n* = 495 (20%) Psychosocial reasons *n* = 268 (10.9%) Catheter dysfunction *n* = 232 (9%) Ultra-filtration failure *n* = 201 (8%) Malnutrition *n* = 70 (3%) Miscellaneous reasons related to PD *n* = 284 (12%) Miscellaneous reasons unrelated to PD *n* = 297 (12%), Unknown reasons *n* = 5, (0.2%)	*n* = 3495 (36%)	*n* = 1489(15%)	N
	HD-PD							
Lobbedez et al., 2013 [27]	PD first	1841	17 m(s)	N	Dialysis adequacy *n* = 493 (25%) Peritonitis *n* = 242 (20%) Psycho-social reasons *n* = 212 (11%) Catheter dysfunction *n* = 183 (9%) Ultrafiltration failure *n* = 162 (6%) Malnutrition miscellaneous *n* = 55(6%)	*n* = 3078 (36%)	*n* = 138 (16%)	N
	HD-PD	183		N	Dialysis adequacy *n* = 40 (22%) Peritonitis *n* = 35 (19%) Psycho-social reasons *n* = 30 (16%) Catheter dysfunction *n* = 21(11%) Ultrafiltration failure *n* = 10 (5%) Malnutrition miscellaneous *n* = 4 (5%) Psychosocial factors linked to the HD-PD dialysis start may affect PD duration.			
Najafi et al., 2012 [28]	PD first	187	N	1 year: 91% 3 years:67% 5 years:41% HD-PD = PD	Peritonitis *n* = 96 (54%) Catheter malfunction *n* = 33 (19%) Patient preference *n* = 31 (17%) Membrane failure *n* = 14 (8%) Exit and tunnel infections *n* = 3 (2%) Mechanical problems *n* = 1 (1%)	*n* = 256 (24%)	*n* = 121 (11%)	Transfer to HD
	HD-PD	49	N		Peritonitis *n* = 30 (61%) Membrane failure *n* = 8 (16%) Patient preference *n* = 8 (16%) Exit and tunnel infections *n* = 2 (4%) Catheter malfunction *n* = 1 (2%) Mechanical problems *n* = 0 (0%)	*n* = 65 (27%)	*n* = 16 (7%)	
Nguyen et al., 2019 [29]	PD first	N	N	30%	N	1 year: 10% 3years: 35% 5 years: 57%	N	N
	HD-PD	N	N	25%	N	1 year: 19% 3years: 46% 5 years: 64% ***		
Pulliam et al., 2014 [30]	PD first	Total: *n* = 350	N	N	N	9%	7%	N
	HD-PD		N	N	N			
Zhang et al., 2013 [31]	PD first	N	54 m(s)	1year:86% 3years:56% 5years:36%	Peritonitis	*n* = 20 1year: 10% 3years: 20%	N	Transfer to HD, died, and transplanted
	HD-PD	N	39 m(s)	1year:74% 3years:41% 5years:18% HD-PD = PD	Peritonitis	*n* = 15 1year: 20% 3years:44%	N	
Zhang et al., 2008 [32]	PD first	Total: *n* = 3	16 ± 16 m(s)	HD-PD<PD	N	*n* = 11 (15%)	*n* = 2 (3%)	N
	HD-PD				N	*n* = 6 (39%)*		

Abbreviations: TF, technique failure TS, technique survival HD, Hemodialysis; PD, Peritoneal dialysis; N, unknown Note: *=*p* < 0.05 **=*p* < 0.01 ***=*p* < 0.001

**Table 3:** Reasons for HD switch to PD

Author (Year)	Number of patients (n)	Reasons for HD transform to PD n (%)
Liberek et al., 2009 [21]	*n* = 67	Vascular access problems Heart failure Intradialytic hypotension
Barone et al., 2014 [22]	*n* = 73	Multiple vascular access failure *n* = 43 (59%) Personal choice *n* = 24 (33%) Cardiovascular disorders *n* = 5 (7%) Living a long distance from the dialysis center *n* = 1 (1%)
Koc et al., 2012 [25]	*n* = 48	Vascular problems *n* = 35 (70%) Patient decision or social problems *n* = 13 (30%)
Najafi et al., 2012 [28]	*n* = 245	Vascular access failure *n* = 110 (45%) Intolerance to HD with intradialytic hypotension *n* = 83 (34%)
Nguyen et al., 2019 [29]	*n* = 911	Patient preference *n* = 581 (64%) Vascular access problems *n* = 60 (7%) Cardiovascular instability *n* = 8 (0.9%) Inadequate solute clearance and fluid removal *n* = 6 (0.7%) Unable to manage self-care *n* = 3 (0.3%) Not recorded *n* = 248 (27%) Others *n* = 5 (0.6%)
Zhang et al., 2008 [32]	*n* = 28	Cardiovascular problems *n* = 16 (6%) Vascular access problems *n* = 6 (21%) Patient choice *n* = 3 (11%) Hemorrhage *n* = 3 (11%)
Zhang et al., 2013 [31]	*n* = 33	cardiovascular disease *n* = 15 (45%) vascular access problems *n* = 8 (24%) patient choice *n* = 5 (15%) others *n* = 5 (15%)

### Technique failure

**Fig. 2:**
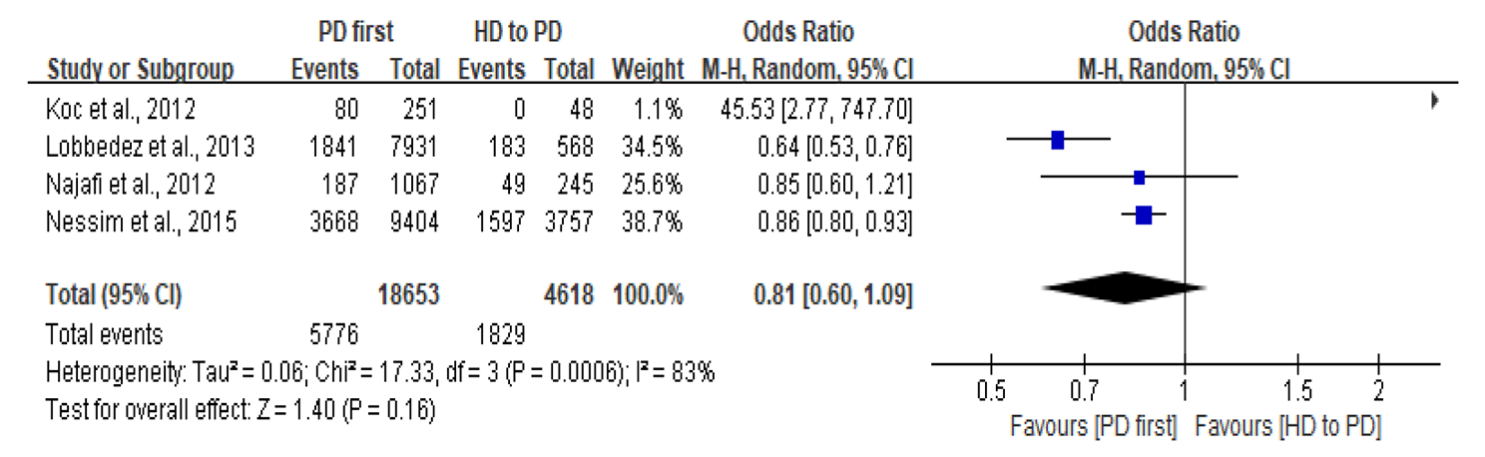
Forest plot of PD dropout for PD first group vs. HD to PD group

TF was defined in six studies [6, 22–24, 28, 31]. TF definitions included a transfer to HD or death [22, 24]; a transfer to HD [6, 23, 28]; and a transfer to HD, transplant, or death [31]. The different definitions of TF are also displayed in Table 2. Based on all definitions of TF, the rate of technique survival at one year varied from 84% to 100. in the PD first group [22–23], and from 74 to 94% in HD to PD group [22, 31]. Four studies found that the PD first group had a better technique survival than the HD to PD group [6, 21, 26, 32], however, only two studies demonstrated a statistically significant difference between the two groups [6, 21]. According to our definition of PD dropout (transfer to HD), four studies were included in the meta-analysis [6, 25, 27, 28]. The meta-analysis found that there was a lower rate of PD dropout in the PD first group, but that there was no statistically significant difference in PD dropout between the two groups (OR: 0.81; 95%CI: 0.61, 1.09; I^2^ = 83%; *P* = 0.16) (Fig. 2).

### The median time on PD

Half studies reported the median time on PD, nevertheless, only three of them outlined the varying durations of PD in two distinct groups [6, 22, 31]. In comparison to patients switching from HD (from 36months to 51months), those who were PD first showed a longer duration on PD (from 48months to 57months) [6, 22].

### The reasons for HD switch to PD

There are seven studies that illustrate the factors leading to the switch from HD to PD [21, 22, 25, 28, 29, 31, 32]. The primary causes for this switch include vascular access failure (ranging from 7 to 70%), patients’ preferences or social issues (ranging from 11 to 64%), and cardiovascular disease (varying from 1 to 45%).

### The reasons for PD dropout

Eight studies reported reasons for PD dropout, however, only three studies presented the reasons and proportion of patients withdrawing from PD in the two groups separately [6, 27, 28]. Inadequate dialysis and peritonitis were the primary reasons for PD dropout in both groups. However, only one study found statistically significant differences in these two reasons between the two groups [6]. Furthermore, Liberek et al. [21] found a correlation between peritonitis and TF only in anuric patients. They discovered that anuric patients transferred from HD to PD have a higher rate of peritonitis than PD first group, resulting in more TF. Reasons for PD dropout also included catheter dysfunction or mechanical problems, ultra-filtration failure, malnutrition, cardiovascular events, patient preference, membrane failure, exit site and tunnel infections [27, 28].

Psychosocial reasons associated with PD dropout were mentioned in three studies. Lobbedez et al. [27] found a higher rate of patients dropping out of PD because of psychosocial reasons in the HD to PD group than PD first group (16% vs. 11%). This was explained by the authors as potentially being due to insecurity and anxiety associated with the change in dialysis modality [26, 27]. They also reported that psychosocial reasons impact the duration of PD for patients transferred from HD. Chidambaram et al. [23] found that patients have 31% higher rate of TF when their physicians are male gender. Suggested reasons for this were more patient-centred communication among female physicians. Dong et al. [24] reported a correlation between the type of medical insurance and the risk of PD dropout in China. They discovered patients in peritoneal dialysis had better technique outcomes whose medical insurance (employee medical insurance) had more reimbursement and wider coverage for the treatment. However, no study reported statistically significant differences between the groups.

### Mortality and transplantation rates

**Fig. 3:**
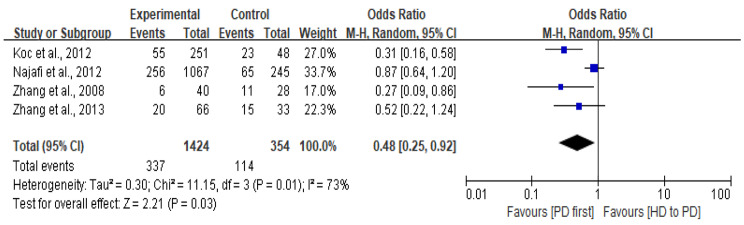
Forest plot of mortality for PD first group vs. HD to PD group

Six studies reported the death rate in each group, and four studies found the HD to PD group had a higher rate of mortality than PD first group that was statistically significant (Table 2) [6, 25, 29, 32]. Four studies reporting the detailed number of deaths were included in the meta-analysis [25, 28, 31, 32]. The meta-analysis demonstrated that patients have a lower mortality rate in the PD first group compared to the HD to PD group (OR: 0.48; 95% CI: 0.25, 0.92; I^2^ = 73%; *P* = 0.03 (Fig. 3)). Furthermore, two studies indicated the number of patients who withdraw from PD for kidney transplantation in the two groups but only one reported a statistically significant difference between the groups (Table 2) [25, 28].

### Quality of included studies and meta-analysis

Two studies did not report the time of follow-up [22, 26]. Three studies did not present confidence intervals [22, 28, 30]. One study lacked detailed information in the methods [32]. No study performed adequate matching for confounding factors. No study included a blinded collection of outcome information.

**Fig. 4:**
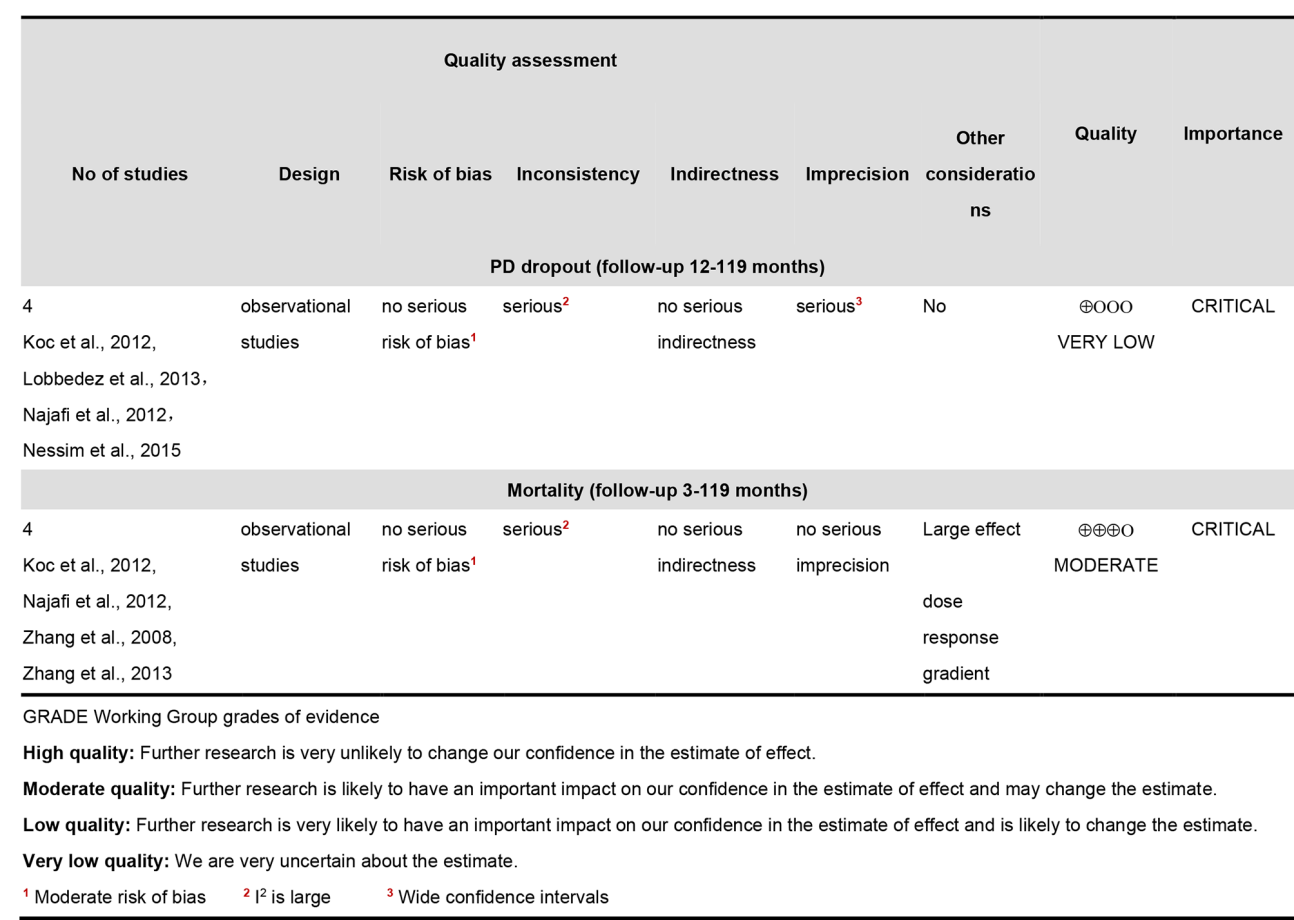
The evidence certainty of meta-analysis

The quality level of the meta-analysis associated with PD dropout was very low due to statistical heterogeneity and wide confidence intervals. The estimates are therefore very uncertain. The meta-analysis of mortality rate had a moderate quality level, indicating the true effects are likely to be close to the estimated effects, but that further research may impact this estimate (Fig. 4).

## Discussion

This systematic review and meta-analysis explored the rates of PD dropout and mortality; as well as the reasons for HD transfer to PD; and PD dropout in HD to PD groups and PD first groups. The research results discovered that PD dropout may not be influenced by previous HD history, but mortality is. The main factors for patient switch from HD to PD are vascular access failure, patients’ preferences or social issues, and cardiovascular disease. Primary reasons for PD dropout are peritonitis and/or inadequate dialysis in both groups. There was no obvious difference between the two groups in the cause of PD dropout.

The main factors impacting patients switch from HD to PD, including vascular access failure, patients’ preferences or social issues, and cardiovascular disease, are in line with the findings of Gallieni et al., [39]. According to Legendre et al., [37] there are two different stages in the switch from HD to PD. During the initial two months, patients’ preferences emerge as the primary reason for transition when patients receive RRT education gradually and have more understanding regarding different dialysis modalities. Whereas in the later stages, the shift is mainly driven by HD-related treatment complications, such as vascular access failure or poor tolerance of HD [6, 38].

Our results demonstrate that previous HD history may not impact the rate of PD dropout. However, a recent systematic review [16] found a higher probability of TF in the HD-PD group than in the PD-first group. Notably, Wang et al. [16] did not uniformly define TF and included only patients who underwent HD for more than 3 months. Legendre et al. [37] suggest that a considerable underestimation of the incidence of HD to PD transfer and its underlying effects may result from excluding early transfers. In contrast, the duration time on HD before switching to PD varied from zero to 284 months in four studies included our meta-analysis [6, 25, 27, 28]. Furthermore, our study has a united definition of PD dropout and only includes studies consistent with our definitions in further meta-analysis. Therefore, excluding patients with early transfer from HD to PD and lack of a unified definition of TF were confounding factors that may have impacted the results of the previous review.

Even though differences in PD dropout are not statistically significant, the mortality rate does significantly differ between the two groups in this meta-analysis. A similar finding was also observed in Wang et al. ‘s meta-analysis [16]. Increased mortality in the HD to PD group may be explained by the difference in residual renal function (RRF) in both groups, which has been reported as an independent prognostic factor to predict mortality [40–42]. Previous studies have demonstrated that HD patients lose RRF significantly faster than PD patients [42, 43]. The drop in RRF ranges from 0.18 to 0.33 ml/min/month in HD patients and from 0.05–0.30 ml/min/month in PD patients [42]. In this systematic review, RRF was reported in four studies. Three of them confirmed that the HD-PD group had statistically significantly lower RRF than the PD first group [25, 31, 32]. HD before switching to PD may negatively affect RRF, so patients may have trouble achieving adequate solute clearances and ultrafiltration on PD and may partly explain the reduced survival as well as the shorter duration time on PD in the HD to PD group [22, 29]. RRF, however, is not always assessed or considered by healthcare professionals in clinical practice, according to Kong, et al. [44]. In all modalities and dialysis prescriptions, Li et al. [40] suggests regular monitoring of RRF.

However, several confounding factors may also explain the correlation between HD history and mortality. Studies have demonstrated that low albumin levels, age over 65, diabetes, employment status, Kt/V ≥ 1.8, ClCr ≥ 50 L/week/1.73 m^2^ are all associated with worse survival outcomes [31, 45, 46]. Furthermore, Legendre et al., [37] argued that it is a high-risk situation when switching between different dialysis modalities, which could influence patients’ survival directly. Jeong et al., [47] also confirmed the association between dialysis modality switch and increased mortality risk based on data from 21,840 dialysis patients.

Peritonitis and/or inadequate dialysis were the main medical reasons for PD dropout in both groups (PD first and HD to PD), according to a wide range of studies conducted in France, Iran, Canada, China, and Italy [6, 26–28, 31]. In one study, the two causes were less common in the PD first group, and this was statistically significant. However, the difference reported between the groups was not particularly substantial (3% vs. 4%) [6]. Wang et al. [16] reported that the peritonitis rate was the same in both groups and similar findings have been identified in this updated review. Interestingly, Workeneh et al. [48] believed that although peritonitis is a common cause of transfer, they stressed patient burnout, noncompliance, inadequate dialysis, and adverse lifestyles may be leading factors contributing to peritonitis.

Notably, dialysis withdrawal reasons may change over time differing from the early stage to later stages. Torres et al. [49] reported that psychosocial reasons account for 100% of early dropouts from PD. Three studies confirmed the impact of psychosocial factors on PD dropout, including gender of physician, insurance type, and anxiety associated with dialysis switching [23, 24, 27]. Apart from these three studies, other evidence has demonstrated numerous psychosocial factors impacting PD dropout, including lack of self-confidence, concerns about appearance, burnout, limitations on social engagement, knowledge of PD and associated treatments, patients’ negative mood, and family dynamics [48, 50]. It is also worth noting that more patient’s dropout of PD for psychosocial reasons in the HD to PD group than in the PD first group (HD<30 days), explained by insecurity and anxiety associated with the change in dialysis modality [27]. Furthermore, psychosocial factors also correlate to the duration time on PD, which may explain the duration time on PD is shorter in HD to PD group than PD first group [27].

Psychosocial factors in PD dropout can be addressed and have been recognized as the most controllable reasons for PD dropout, especially for early dropout [49, 51]. It is suggested multidisciplinary teams take psychosocial factors into consideration to determine which modifiable PD center characteristics can help to reduce insecurity, burnout, and anxiety relevant to dialysis. This includes considering staffing and caregiver training programs, infection control protocols and practices, and quality improvement activities, while also noting that HD to PD groups may require more psychosocial support than the PD first group [24, 50, 52, 53].

### Strengths and limitations

This study has several strengths. First, we have used a definition of “PD dropout” that was uniform across studies subjected to meta-analysis. In screening articles for inclusion in the meta-analysis, we included articles that met the definition of dropout for this study, thus reducing confounding factors due to the diversity of definitions. Secondly, there was no restriction on HD time before transferring to PD in this study, which was suggested by Legendre et al. [37] to avoid the underestimation of the incidence of the transfer from HD to PD. Finally, we assessed the quality of the meta-analysis using the GRADE tool to better reflect the overall quality of the evidence presented and the value of including this data in future clinical guidance [54]. This tool showed a very low to moderate level of quality, indicating uncertainty in the evidence. Furthermore, the participants in the study had different characteristics at baseline. This was reported fully in our data tables that display and report any inconsistency of the patients included in studies at baseline. This inconsistency may have confounded the results and is an important consideration when applying this evidence to clinical practice.

There are also some limitations of this study. Varied definitions of PD dropout across different studies introduce a potential bias that could influence the research outcomes. Furthermore, the quality of the meta-analysis appears to be low, as indicated by the GRADE assessment.

## Conclusion

This study confirmed that HD history may not impact PD dropout rates but could impact mortality. There were no significant differences in reasons of PD dropout in PD first and HD to PD groups. However, the impact of psychosocial reasons remains a gap in the current evidence base. Further research is required to investigate the psychosocial differences between the HD to PD group and PD first group. Importantly, we have provided strong clinical evidence and suggestions for healthcare professionals based on the study results. In the future, multidisciplinary training and dialysis education programs could be developed to emphasize the importance of RRF assessments and evaluate the impact of providing more psychosocial support to HD patients transferring to PD. There is also a need for a consensus on the definition of PD dropout to establish a standard for future research.

### Electronic supplementary material

Below is the link to the electronic supplementary material.

Supplementary Material 1

Supplementary Material 2

## Data Availability

The datasets used and/or analysed during the current study available from the corresponding author on reasonable request.

## Abbreviations

CASP: Critical Appraisal Skills Programme
TF: Technique Failure
TS: Technique Survival
HD: Haemodialysis
PD: Peritoneal dialysis
RRF: Residual renal function
