# Supplementary material for: Rate and reasons for peritoneal dialysis dropout following haemodialysis to peritoneal dialysis switch: a systematic review and meta-analysis
Source: BMC Nephrol. 2024 Mar 16;25:99. doi: 10.1186/s12882-024-03542-w (PMC10943899; doi:10.1186/s12882-024-03542-w)
Supplement: Supplementary file 1 — Supplementary Material 1 [file 12882_2024_3542_MOESM1_ESM.docx]

| **Appendix 1 The scores of CASP assessments**  CASP Criteria | Barone et al., 2014 | Chidambaram et al., 2011 | Dong et al., 2022 | Koc et al., 2012 | Liberek et al., 2009 | Lobbedez et al., 2012 | Lobbedez et al., 2013 | Najafi et al., 2012 | Nessim et al., 2015 | Nguyen et al.2019 | Pulliam et al., 2014 | Zhang et al., 2013 | Zhang et al., 2008 |
| --- | --- | --- | --- | --- | --- | --- | --- | --- | --- | --- | --- | --- | --- |
| 1.Did the study address a clearly focused issue? | 2 | 2 | 2 | 2 | 2 | 2 | 2 | 2 | 2 | 2 | 2 | 2 | 2 |
| 2.Was the cohort recruited in an acceptable way? | 2 | 2 | 2 | 2 | 2 | 2 | 2 | 2 | 2 | 2 | 2 | 2 | 2 |
| 3.Was the exposure accurately measured to minimise bias? | 2 | 2 | 2 | 2 | 2 | 2 | 2 | 2 | 2 | 2 | 2 | 2 | 1 |
| 4.Was the outcome accurately measured to minimise bias? | 1 | 0 | 1 | 1 | 1 | 1 | 1 | 1 | 1 | 1 | 1 | 1 | 1 |
| 5.Have the authors identified all important confounding factors?  Have they taken account of the confounding factors in the design and/ or analysis? | 0 | 0 | 0 | 0 | 0 | 0 | 0 | 0 | 0 | 0 | 0 | 0 | 0 |
| 6.Was the follow up of subjects complete enough?  Was the follow up of subjects long enough? | 1 | 2 | 2 | 2 | 2 | 1 | 2 | 2 | 2 | 2 | 2 | 2 | 2 |
| 7.What are the results of this study? | 2 | 2 | 2 | 2 | 0 | 2 | 2 | 2 | 2 | 2 | 2 | 2 | 2 |
| 8.How precise are the results? | 1 | 2 | 2 | 2 | 2 | 2 | 2 | 1 | 2 | 2 | 2 | 2 | 1 |
| 9.Do you believe the results? | 2 | 2 | 2 | 2 | 2 | 2 | 2 | 2 | 2 | 2 | 2 | 2 | 2 |
| 10.Can the results be applied to the local population? | 2 | 2 | 2 | 2 | 2 | 2 | 2 | 2 | 2 | 2 | 2 | 2 | 2 |
| 11.Do the results of this study fit with other available evidence? | 2 | 2 | 2 | 2 | 2 | 2 | 2 | 2 | 2 | 2 | 2 | 2 | 2 |
| 12.What are the implication of this study for practice? | 2 | 2 | 2 | 2 | 2 | 2 | 2 | 2 | 2 | 2 | 2 | 2 | 2 |
| Total score of 24 | 21 | 20 | 21 | 21 | 20 | 20 | 20 | 20 | 21 | 21 | 21 | 21 | 19 |
